# Cabozantinib inhibits AXL- and MET-dependent cancer cell migration induced by growth-arrest-specific 6 and hepatocyte growth factor

**DOI:** 10.1016/j.bbrep.2020.100726

**Published:** 2020-01-17

**Authors:** Takahito Hara, Akiko Kimura, Tohru Miyazaki, Hiroshi Tanaka, Megumi Morimoto, Katsuhiko Nakai, Junpei Soeda

**Affiliations:** a Innovation Promotion, Shonan Research Central Office, Research, Takeda Pharmaceutical Company Limited, 2-26-1 Muraoka-Higashi, Fujisawa-shi, Kanagawa, 251-8555, Japan; b Oncology Therapeutic Area Unit for Japan & Asia, Takeda Pharmaceutical Company Limited, 4-1-1 Dosho-machi Chuo-ku Osaka-shi, Osaka, 540-8645, Japan; c Department of Japan Medical Affairs, Japan Oncology Business Unit, Takeda Pharmaceutical Company Limited, 2-1-1 Nihonbashi-Honcho, Chuo-ku, Tokyo, 103-8668, Japan; d Axcelead Drug Discovery Partners, Inc., 2-26-1Muraoka-Higashi, Fujisawa-shi, Kanagawa, 251-0012, Japan

**Keywords:** Growth arrest-specific 6, Hepatocyte growth factor, AXL receptor tyrosine kinase, *MET* proto-oncogene-encoded receptor tyrosine kinase, Cabozantinib, Cancer cell migration

## Abstract

Cabozantinib is known as an inhibitor of receptor tyrosine kinases mainly targeting AXL receptor tyrosine kinase (AXL), *MET* proto-oncogene-encoded receptor tyrosine kinase (MET), and vascular endothelial growth factor receptor 2. Growth arrest-specific 6 (GAS6) and hepatocyte growth factor (HGF), the natural ligands of AXL and MET, respectively, are associated with the induction of cancer cell proliferation or metastasis. Currently, it is still unclear how cabozantinib regulates cancer cell migration and invasion by inhibiting AXL and MET. This study was conducted to investigate the mechanism underlying the anti-cancer effects of cabozantinib through regulation of AXL and MET signaling.

The results of Boyden chamber assays showed that cancer cell migration was induced by GAS6 and HGF in SKOV3 cells in serum-free medium. Combinatorial treatment with GAS6 and HGF exerted an additive effect on cell migration. Furthermore, we examined the role of AXL and MET signaling in cell migration. Short interfering RNA targeting AXL and MET inhibited GAS6- and HGF-induced migration, respectively. Double knockdown of AXL and MET completely suppressed cell migration induced by combination treatment with GAS6 and HGF compared to AXL or MET inhibition alone. Finally, we investigated the effects of cabozantinib on cell migration and invasion. Cabozantinib inhibited AXL and MET phosphorylation and downregulated the downstream mediators, phosphorylated SRC in the presence of both GAS6 and HGF in SKOV3 cells. The cell migration and invasion induced by combined GAS6 and HGF treatment were suppressed by cabozantinib, but not by capmatinib, a selective MET inhibitor.

Our data indicate that the GAS6-AXL and HGF-MET signal pathways markedly contribute to cancer cell migration and invasion in an independent manner, suggesting that simultaneous inhibition of these two pathways contributes to the anti-cancer effects of cabozantinib.

## Highlights

• Response to GAS6 and HGF for cell migration differs between cancer cell lines.
• GAS6-AXL and HGF-MET pathways independently contribute to cancer cell migration.
• Cabozantinib inhibits both GAS6-AXL and HGF-MET pathways in cancer cells.
• Cabozantinib inhibits GAS6 and HGF combination-induced cancer cell migration.
• Cabozantinib inhibits GAS6 and HGF combination-induced cancer cell invasion.

## 1 Introduction

Cabozantinib is a small-molecule tyrosine kinase inhibitor (TKI) that uniquely inhibits the phosphorylation of AXL, MET, and vascular endothelial growth factor receptor 2 (VEGFR2) along with RET and ROS1 [1,2]. This compound, as a single agent, has been approved in several countries or regions including the US and the EU for treating progressive metastatic medullary thyroid carcinoma, advanced renal cell carcinoma (RCC), and recently, hepatocellular carcinoma.

*MET* proto-oncogene-encoded receptor tyrosine kinase (MET) is a receptor tyrosine kinase (RTK) expressed on the surfaces of various epithelial cells and binds to its ligand hepatocyte growth factor (HGF) [3]. Genetic alternations and overexpression of MET are broadly observed in various cancer types such as lung cancer, breast cancer and glioblastoma, and abnormal activation of HGF-MET signaling is involved in tumor progression and metastasis [4,5]. Furthermore, increased MET expression is associated with poor prognosis and resistance to targeted therapies in patients with RCC, ovarian cancer, and non-small-cell lung cancer [[6], [7], [8], [9]].

AXL, another target molecule of cabozantinib, belongs to the TAM (Tyro3, AXL, and Mer) RTK family. Growth arrest-specific 6 (GAS6) is the common ligand of TAM family members [10]. The GAS6-AXL pathway plays critical roles in regulating cell survival, proliferation, invasion, migration, and immune function [10,11]. Overexpression or activation of AXL has been clinically linked to high invasiveness and metastasis in various cancers [[12], [13], [14], [15]]. AXL knockdown (KD) by short interfering RNA (siRNA) reduced cell viability and down-regulated PI3K/AKT signaling in RCC cells [16]. It has also been reported that AXL and MET are involved in acquired resistance to sunitinib and that cabozantinib has anti-tumor activity against sunitinib-resistant cells both *in vitro* and *in vivo* [17].

Many molecular targeting agents, especially TKIs, have been developed for cancer treatment so far. Their efficacy profiles are different based on the mode of actions. We hypothesized robust clinical efficacy of cabozantinib is derived from the combined targeted inhibition against key signaling for proliferation/survival/mobilization of tumor cells. At present, it remains unclear how cancer cell migration and invasion are regulated by cabozantinib via inhibition of AXL and MET. In this study, we investigated the mechanism underlying the anti-cancer effects of cabozantinib through the regulation of GAS6-AXL and HGF-MET signaling.

## 2 Materials and Methods

### 2.1 Cell culture and reagents

The human cancer cell lines, SKOV3 (ovarian cancer), PC3 (prostate cancer), HT1080 (fibrosarcoma), and NCI-H522 (lung cancer) were purchased from the American Type Culture Collection (Manassas, VA, USA). Cell lines were cultured in McCoy's medium (SKOV3), EMEM medium (HT1080), or RPMI-1640 medium (PC3 and NCI-H522) supplemented with 10% fetal bovine serum at 37 °C in 5% CO_2_. GAS6 and HGF were purchased from R&D Systems (Minneapolis, MN, USA) and PeproTech, (Rocky Hill, NJ, USA), respectively. Cabozantinib and Capmatinib were purchased from MedChemExpress (Monmouth Junction, NJ, USA). Antibodies specific for pAXL (Tyr702) (#5724), AXL (#8661), pMET (Tyr1234/1235) (#3077), pAKT (Ser473) (#9271), AKT (#9272), SRC (#2109), phosphorylated extracellular signal-regulated kinase (pERK) (Thr202/Tyr204) (#9101), ERK (#9102), and GAPDH (#3683) were purchased from Cell Signaling Technology (Danvers, MA, USA) and MET (71–8000) and pSRC (Tyr418) (44-660G) were purchased from Thermo Fisher Scientific (Waltham, MA, USA).

### 2.2 Immunoblotting

Whole-cell lysates were obtained by lysing the cells in buffer containing 62.5 mM Tris-HCl, 10% glycerin, 2% SDS, protease inhibitor (Roche, Basel, Switzerland), and phosphatase inhibitor (Roche). The protein concentration was determined with a BCA assay kit (Thermo Fisher Scientific). Proteins separated by SDS-PAGE were transferred onto polyvinylidene fluoride membranes (Bio-Rad, Hercules, CA, USA). Membranes were blocked with PVDF blocking buffer (TOYOBO, Osaka, Japan) and then probed with primary antibodies diluted in Can Get Signal I solution (TOYOBO) overnight at 4 °C. Incubation was then performed with secondary antibodies diluted in Can Get Signal II solution (TOYOBO). The bands were detected with ECL Select (Amersham plc, Amersham, UK) according to the manufacturer's protocol. The intensity of bands was detected with an ImageQuant LAS 4000 mini (GE Healthcare, Little Chalfont, UK) and analyzed with ImageQuant TL software.

### 2.3 Migration/invasion assay

Cell migration and invasion assays were performed using a CytoSelect 24-well cell migration assay and CytoSelect 24-well invasion assay, respectively, 8-μm pore size Boyden chamber plate (Cell Biolabs, San Diego, CA, USA) according to the manufacturer's protocol. Briefly, the cells were incubated in serum-free medium for 24 h and the cell suspension (1.25 ⨯ 10^5^ cells for migration, 3.3 ⨯ 10^5^ cells for invasion) was plated on the upper chamber. Serum-free medium containing 100 ng/mL of GAS6, 100 ng/mL of HGF, or both (each 100 ng/mL) was added to the lower chamber. After a 24 h, migratory or invasive cells were lysed and dyed with CyQuant GR fluorescent dye. Fluorescence was determined with a fluorimeter at 485/535 nm. For compound treatments, 0.1 or 1 μM of cabozantinib or capmatinib was added to both chambers. We confirmed the growth of SKOV3 cells was not affected by these compounds at 1 μM (data shown).

### 2.4 siRNA-mediated gene silencing

siRNA transfection was conducted using Lipofectamine RNAiMAX Reagent (Thermo Fisher Scientific) according to the manufacturer's protocol and Silencer Select siRNAs (Thermo Fisher Scientific) were used to silence the AXL and MET genes in SKOV3 cells. siRNA transfection was carried according to the manufacturer's protocol at a final concentration of 5 nM. For double knockdown, both siRNAs were used. The concentration of siRNA was normalized using negative control siRNA. Knockdown was evaluated at 72 h by immunoblotting.

### 2.5 Statistical analysis

Statistical analyses were performed using Dunnett's or Student *t* tests (EXSUS ver.8, CAC Croit Corporation, Tokyo, Japan). A difference of *P* < 0.05 was considered to indicate statistically significance.

## 3 Results

**Fig. 1:**
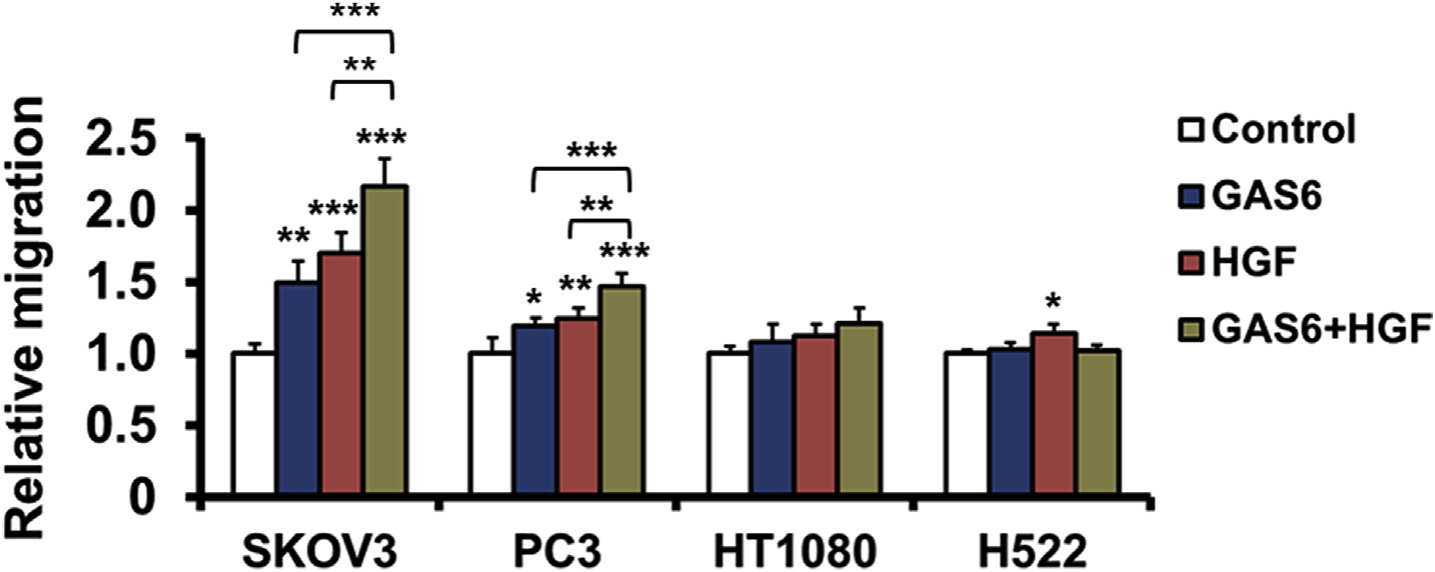
Induction of cell migration by GAS6 and HGF in cancer cell lines. SKOV3, PC3, HT1080, and H522 cells were serum-starved overnight and then seeded into the upper chambers of Boyden chambers in serum-free medium, with GAS6 (100 ng/mL), HGF (100 ng/mL), or a combination of GAS6 (100 ng/mL) and HGF (100 ng/mL) in serum-free medium in the lower chambers. After 24 h, cells that had migrated to the lower chamber were enumerated as described in the Materials and Methods. The data represent mean values. Bars, +SE. n = 3–4. *, P < 0.05; **, P < 0.01; ***, P < 0.001 versus GAS6/HGF (−) serum-free medium control, unless otherwise indicated, by Dunnett's test.

To determine whether GAS6 and HGF stimulate cell migration, we conducted Boyden chamber assays using serum-free media. Cells were seeded onto the upper chamber, and GAS6, HGF, or a combination of GAS6 and HGF was added to the lower chamber. We used SKOV3, PC3, HT1080, and NCI-H522 cells, all of which were confirmed to have migratory potential according to Boyden chamber assays conducted using 10% FBS as a chemoattractant (data not shown). Among the four cell lines, SKOV3 and PC3 cells responded to GAS6 and HGF (*P = 0.0011* for GAS6, *P < 0.0001* for HGF, *P < 0.0001* for GAS6+HGF in SKOV3. *P = 0.0196* for GAS6, *P = 0.0033* for HGF, *P < 0.0001* for GAS6+HGF in PC3. Fig. 1). Combined treatment with GAS6 and HGF promoted cell migration to a greater extent than each treatment alone in both cell lines (GAS6+HGF vs GAS6; *P = 0.004*, GAS6+HGF vs HGF; *P = 0.0053* in SKOV3. GAS6+HGF vs GAS6; *P = 0.0008*, GAS6+HGF vs HGF; *P = 0.0039* in PC3, Fig. 1). Because SKOV3 cells showed a better response to both GAS6 and HGF compared to PC3 cells, we selected SKOV3 cells for subsequent studies.

**Fig. 2:**
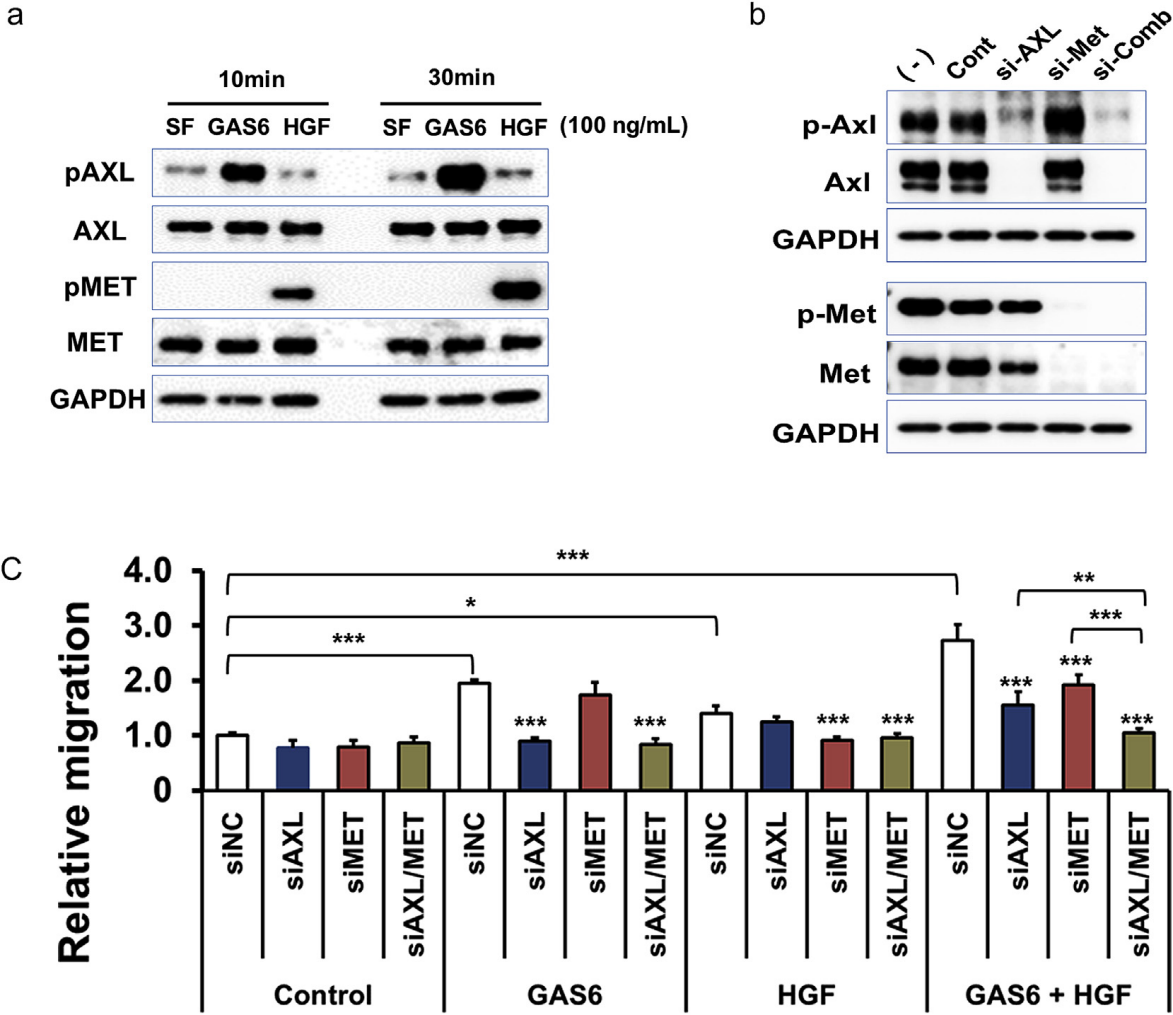
GAS6- and HGF-induced migration depends on AXL and MET, respectively, in SKOV3 cells. A. Phosphorylation of AXL and MET was induced by GAS6 and HGF, respectively, in SKOV3 cells. Cells were treated with GAS6 (100 ng/mL) or HGF (100 ng/mL) for 10 and 30 min after serum starvation overnight. AXL, pAXL, MET, and pMET levels were determined via immunoblot analysis. B. Knockdown of AXL and MET via siRNA in SKOV3 cells. Cells were treated with siRNAs against AXL, or MET, or AXL and MET together for 72 h and AXL, pAXL, MET, and pMET levels were determined via immunoblot analysis. C. KD of AXL or MET individually and in combination suppressed GAS6-, HGF-, and combinatorial GAS6- and HGF-induced cell migration to baseline levels, respectively. Cells were treated with siRNA via reverse transfection for 24 h, followed by an additional 24 h of serum starvation. Thereafter, cells were plated into the upper chambers of Boyden chambers in serum-free medium, with GAS6 (100 ng/mL), HGF (100 ng/mL), or both GAS6 (100 ng/mL) and HGF (100 ng/mL) in serum-free medium in the lower chambers. After 24 h, the cells that had migrated to the lower chambers were enumerated as described in the Materials and Methods. The data represent the mean values. Bars, +SE. n = 4. **, P < 0.01; ***, P < 0.001 vs siNC (negative control), unless otherwise indicated, by Dunnett's test.

GAS6 and HGF activated their cognate receptors AXL and MET, respectively, in SKOV3 cells. GAS6 treatment induced the phosphorylation of AXL but not MET in SKOV3 cells (Fig. 2A). In contrast, HGF induced MET phosphorylation, but not that of AXL (Fig. 2A). To determine whether GAS6-AXL and HGF-MET signaling were necessary to stimulate cell migration, we knocked down *AXL* and *MET* using siRNA. siRNA targeting AXL downregulated AXL and phosphorylated AXL (pAXL) levels after 72 h of transfection (Fig. 2B). KD of AXL tended to decrease total MET levels (Fig. 2B), but it did not affect phosphorylated MET (pMET) levels (Fig. 2B). siRNA targeting MET downregulated MET and pMET levels after 72 h of transfection, while it did not affect pAXL (Fig. 2B). Combined treatment with siRNAs targeting *AXL* and *MET* downregulated AXL, pAXL, MET, and pMET (Fig. 2B). siRNA-treated cells were seeded into the upper chamber, and GAS6, HGF, or a combination of GAS6 and HGF was added to the lower chamber. KD of AXL suppressed GAS6-induced migration to baseline levels (*P < 0.0001*), but not HGF-induced cell migration (*P = 0.104,* Fig. 2C). KD of MET suppressed HGF-induced cell migration to baseline levels (*P < 0.0001*), but not GAS6-induced cell migration (*P = 0.0850*, Fig. 2C). Using a combination of GAS6 and HGF as chemoattractants, simultaneous KD of AXL and MET suppressed cell migration to baseline levels (*P < 0.0001*) and to a larger extent than with KD of AXL or MET alone (siAXL+siMET vs siAXL; *P = 0.0072*, siAXL+siMET vs siMET; *P = 0.0002*, Fig. 2C). These findings indicate that cell migration induced by GAS6 and HGF depends on AXL and MET signaling, respectively, in an independent manner in SKOV3 cells.

**Fig. 3:**
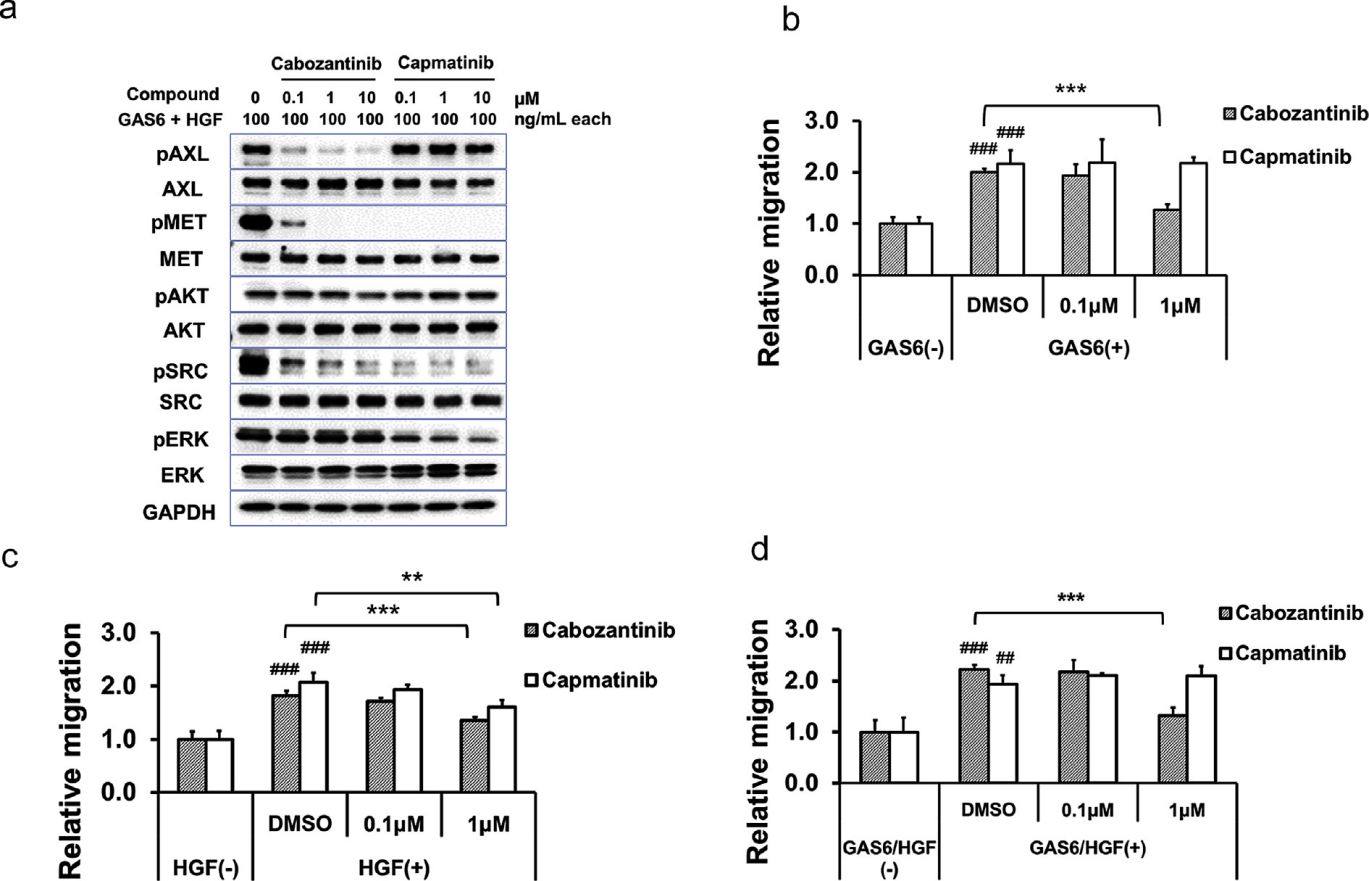
GAS6- and HGF-induced migration was inhibited by cabozantinib in SKOV3 cells. A. Cells were serum-starved overnight and then treated with cabozantinib (0.1, 1, and 10 μM) or capmatinib (0.1, 1, and 10 μM) for 10 min, followed by treatment with GAS6 (100 ng/mL) and HGF (100 ng/mL) together for 10 min. AXL, pAXL, MET, pMET, AKT, pAKT, SRC, pSRC, ERK, and pERK levels were determined via immunoblot analysis. B –D. Cells were serum-starved overnight and then seeded into the upper Boyden chambers in serum-free medium with serum-free medium, GAS6 (100 ng/mL) (B), HGF (100 ng/mL) (C), or both GAS6 (100 ng/mL) and HGF (100 ng/mL) (D) in the lower chambers. DMSO, cabozantinib (0.1 and 1 μM) or capmatinib (0.1 and 1 μM) were added to both the upper and lower chambers. After 24 h, cells that had migrated to the lower chambers were enumerated as described in the Materials and Methods. The data represent the mean values. Bars, +SE. n = 3–4. **, P < 0.01; ***, P < 0.001 vs DMSO control by Dunnett's test. ##, P < 0.01; ###, P < 0.001 vs GAS6/HGF (−) control by Student *t*-test.

Cabozantinib is a multi-target RTK inhibitor that primarily inhibits AXL, MET, and VEGFR2 [1]. We performed a migration assay using SKOV3 cells to determine whether cabozantinib suppresses GAS6-induced or HGF-induced cell migration. First, we confirmed whether cabozantinib inhibits AXL and MET signals in SKOV3 cells. Cabozantinib treatment inhibited both AXL and MET phosphorylation in a dose-dependent manner with total inhibition at ≥1 μM when SKOV3 cells were simultaneously stimulated with GAS6 and HGF (Fig. 3A). pAKT, pSRC, or pERK are downstream mediators of AXL and MET signaling [3,10]. Cabozantinib treatment decreased pSRC levels at ≥0.1 μM, but not pAKT and pERK levels even at 10 μM in SKOV3 cells (Fig. 3A). Capmatinib, a selective MET inhibitor, suppressed MET phosphorylation at ≥0.1 μM, but not AXL phosphorylation even at 10 μM (Fig. 3A). However, capmatinib suppressed both ERK and SRC phosphorylation at ≥0.1 μM in SKOV3 cells (Fig. 3A).

Next, we investigated the effect of cabozantinib and capmatinib on cell migration with GAS6, or HGF, or combination of GAS6 and HGF as chemoattractants for SKOV3 cells. Cabozantinib inhibited cell migration under all conditions at 1 μM (*P = 0.0001* for GAS6, *P < 0.0001* for HGF, *P = 0.0001* for GAS6+HGF Fig. 3B–D). Capmatinib inhibited HGF-induced cell migration (*P = 0.0020* Fig. 3C) but did not suppress GAS6-induced or combined GAS6- and HGF-induced migration at 1 μM (*P = 0.9980* for GAS6, *P = 0.2434* for GAS6+HGF Fig. 3B, D).

**Fig. 4:**
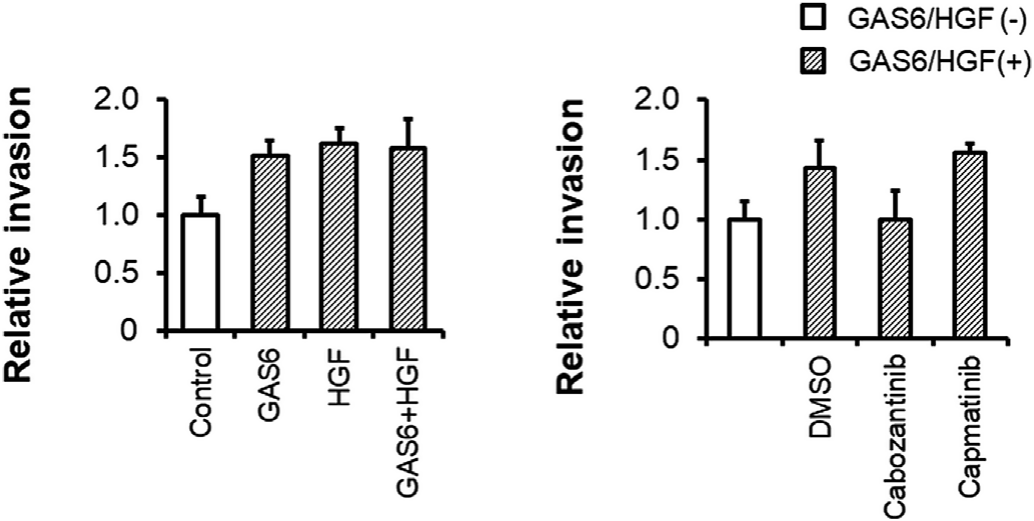
GAS6- and HGF-induced invasion was inhibited by cabozantinib in SKOV3 cells. A. Cells were serum-starved overnight and then seeded into the upper Boyden chambers in serum-free medium with serum-free medium, GAS6 (100 ng/mL), HGF (100 ng/mL), or combination of GAS6 (100 ng/mL) and HGF (100 ng/mL) in the lower chambers. *, P < 0.05; **, P < 0.01 vs GAS6/HGF (−) control by Dunnett's test. B. Cells were serum-starved overnight and then seeded in the upper Boyden chambers in serum-free medium with serum-free medium with combination of GAS6 (100 ng/mL) and HGF (100 ng/mL) in the lower chambers. DMSO, cabozantinib (1 μM) or capmatinib (1 μM) were added to both the upper and lower chambers. After 24 h, cells invasion into the lower chambers was enumerated as described in the Materials and Methods. The data represent the mean values. Bars, +SE. n = 3–4. *, P < 0.05; **, P < 0.01 vs GAS6/HGF (−) control by Dunnett's test. #, P < 0.05 by Student *t*-test.

Finally, we investigated the effect of cabozantinib and capmatinib on cell invasion using a combination of GAS6 and HGF as chemoattractants for SKOV3 cells. GAS6, HGF, and the combination of GAS6 and HGF stimulated cell invasion (*P = 0.0159* for GAS6, *P = 0.0062* for HGF, *P = 0.0084* for GAS6+HGF Fig. 4A). Consistent with the findings of cell migration analysis, 1 μM cabozantinib, but not capmatinib, inhibited cell invasion following combination induction by GAS6 and HGF (*P = 0.0247* for cabozantinib, *P = 0.5557* for capmatinib, Fig. 4B).

## 4 Discussion

This study shows that cell migration was induced by GAS6 and HGF in some cancer cell lines but not in others through Boyden chamber assays. Using SKOV3 cells which are responsive to GAS6 and HGF, we found that 1) combined GAS6 and HGF exerted an additive effect on cell migration, 2) AXL and MET signaling is necessary for GAS6- and HGF-induced migration, respectively, 3) simultaneous gene silencing of AXL and MET, but not AXL or HGF alone, completely suppressed cell migration induced by combination treatment with GAS6 and HGF, and 4) cell migration and invasion induced by a combination of GAS6 and HGF was suppressed by cabozantinib inhibition of both AXL and MET, but not by capmatinib which selectively inhibited MET.

GAS6-AXL signaling is associated with cancer cell invasiveness and migration [10]. Our results showed that GAS6 stimulated cell migration in SKOV3 and PC3 cells but not in HT1080 and NCI-H522 cells. AXL expression and cancer cell invasiveness were confirmed via Boyden chamber assays using serum as a chemoattractant for the four cell lines (data not shown).

The concentration of GAS6 and HGF (100 ng/mL) used in this study was similar to clinically observed concentration. It has been reported that plasma GAS6 concentrations were 52.0 ng/mL and 63.8 ng/mL in healthy men and women, respectively [18]. Local GAS6 concentrations in the tumor microenvironment (TME) may be higher [19].

The TME is comprised of various cells including mesenchymal cells, adipocytes, vascular endothelial cells, blood cells, and immune cells [11]. Therefore, tumor cells are potentially exposed to various growth factors and cytokines in an autocrine and paracrine manner in the TME. Our results revealed that GAS6 and HGF had an additive effect on cell migration, suggesting that cancer progression is promoted by various humoral factors such as GAS6 and HGF in the TME. Furthermore, our results show that simultaneous inhibition of AXL and MET exerted superior inhibitory effects on cancer cell migration compared to individual inhibition, suggesting that combinatorial inhibition of various oncogenic signals effectively suppresses cancer progression.

In Boyden chamber assays for cancer cells, serum-containing medium is placed in the lower chamber, with serum serving as a chemoattractant in many studies. One study reported the inhibitory effect of cabozantinib and AXL or MET KD on the invasion of RCC cells under such conditions [17]. However, it is unclear which factors in the serum induce cell invasion or migration under such conditions. In this study, we conducted cell migration and invasion assays under serum-free conditions, using GAS6 or HGF as chemoattractants to dissect the roles of AXL and MET in cabozantinib-mediated inhibition of cancer cell migration and invasion. siRNA-mediated KD revealed that GAS6- and HGF-induced migration solely depended on AXL and MET signaling, respectively, in SKOV3 cells. Cabozantinib suppressed GAS6- and HGF-induced cell migration and invasion in SKOV3 cells, suggesting that the inhibition of cell migration and invasion by cabozantinib is mediated through AXL and MET inhibition. It should be noted that SKOV3 cells is reported to express Mer and Tyro3 [20]. GAS6 is not only the ligand of AXL, but also the ligand of Mer and Tyro3. These molecules are known to contribute to migration and invasion of cancer [21]. Our data demonstrates that AXL pathway, rather than Mer and Tyro3 pathways, is critical for cell migration at least in SKOV3 cells.

The critical role of GAS6-AXL signaling in prostate cancer cell invasion was previously reported using shRNA targeting AXL in Boyden chamber assays under serum-free conditions with GAS6 as a chemoattractant [22]. Michael et al. reported that cabozantinib inhibits HGF-stimulated migration and invasion of melanoma cells in Boyden chamber assays when HGF was used as a chemoattractant under serum-free conditions [1]. Together with these reports, our findings suggest that HGF and GAS6 induce migration and invasion in various types of cancer, and cabozantinib suppresses cancer cell migration and invasion by inhibiting AXL and MET.

In this study, cabozantinib significantly inhibited GAS6- and HGF-induced cell migration and invasion at a concentration of 1 μM. The mean steady-state plasma level in patients with medullary thyroid cancer administered an effective dose of 140 mg cabozantinib was 1380 ng/mL (2.7 μM) [23]. Therefore, our findings may reflect the clinical setting.

We demonstrated that GAS6 stimulated cell migration in PC3 cells. In PC3 cells, we have obtained the data that an invasive subline of PC3 cells had higher levels of pAXL than a non-invasive subline, where the invasive and non-invasive sublines were established by collecting the fraction that invaded under or remained on the Matrigel membrane of Boyden chambers, respectively (data not shown). The invasion of the invasive subline was suppressed by cabozantinib (data not shown). These findings further support the critical role of AXL in cancer invasion and the effectiveness of cabozantinib.

Recent studies reported that MET interacts with AXL [[24], [25], [26]]. Upon interacting with HGF, MET promotes AXL phosphorylation to activate RAC1-dependent cell migration and invasion [25]. In contrast, AXL interacts with GAS6 to stimulate SRC phosphorylation to activate MET and maximize cell invasion [24]. However, in this study, interactive signaling between the GAS-AXL and HGF-MET axes was not clearly observed. KD of AXL did not markedly influence pMET levels and vice versa. KD of AXL tended to decrease total MET levels, but the reason is unclear at present. Furthermore, KD of AXL did not suppress HGF-induced migration and vice versa. Therefore, we did not investigate the interaction between AXL and MET molecules through immunoprecipitation assays. Interactive signaling between AXL and MET may be cell context-dependent.

There were some limitations to this study. First, downstream signaling responsible for AXL- and MET-mediated migration was unclear; pAKT, pSRC, or pERK did not fully account for the cabozantinib- and capmatinib-mediated inhibition of cell migration in SKOV3 cells. Second, our results revealed the inhibitory effects of cabozantinib on GAS6- and HGF-dependent cell migration and invasion only *in vitro*. Genetic inactivation of *AXL* or soluble AXL receptors prevents the metastasis of SKOV3-derived cells *in vivo* [27]. Furthermore, cabozantinib decreases MET, AXL, and AKT phosphorylation in colorectal cancer patient-derived tumor xenografts [28]. Further studies are required to confirm these results.

In summary, our data show that the GAS6-AXL and HGF-MET signaling axes play a critical role in cancer cell migration and invasion, demonstrating that simultaneous inhibition of these pathways contributes to the anti-cancer effects of cabozantinib.

## Funding

All experiments performed in this study were funded by Takeda Pharmaceutical Company Limited.

## Author contribution

T.H., A.K., T.M., H.T. and M.M. conceived and planned the experiments. H.T. and M.M. carried out the experiments. T.H., A.K., T.M., H.T., and M.M. contributed to the interpretation of the results. T.H. wrote the main manuscript. A.K., K.N., and J.S. supervised the project. All authors provided critical feedback and helped shape the research, analysis and manuscript.

## CRediT authorship contribution statement

**Takahito Hara:** Conceptualization, Methodology, Validation, Resources, Writing - original draft, Writing - review & editing, Project administration. **Akiko Kimura:** Conceptualization, Methodology, Writing - review & editing, Supervision. **Tohru Miyazaki:** Methodology, Writing - original draft, Writing - review & editing, Project administration. **Hiroshi Tanaka:** Methodology, Validation, Formal analysis, Investigation, Resources, Data curation, Writing - original draft, Writing - review & editing, Visualization. **Megumi Morimoto:** Methodology, Validation, Formal analysis, Resources, Data curation, Writing - review & editing, Visualization. **Katsuhiko Nakai:** Conceptualization, Writing - review & editing, Supervision. **Junpei Soeda:** Conceptualization, Writing - review & editing, Supervision.

## Declaration of competing interest

T.H., A.K., T.M, MM, K.N and J.S. are employed by Takeda; H.T. is employed by Axcelead.
